# Hepatincolaceae (Alphaproteobacteria) Symbionts of Snapping Shrimp *Alpheus brevicristatus*: Genomic Capacity for Functions Beyond Nutrient Scavenging

**DOI:** 10.3390/microorganisms14081864

**Published:** 2026-08-21

**Authors:** Fang-Chao Zhu, Yan-Bin Yang, Pei-Pei Liu, Xin Liu, Qun-Jian Yin, Xu-Yang Chen, Shuo Yu

**Affiliations:** 1 Key Laboratory of Tropical Marine Ecosystem and Bioresource, Fourth Institute of Oceanography, Ministry of Natural Resources, Beihai 536015, China; zhufangchao@4io.org.cn (F.-C.Z.); 13557520175@163.com (Y.-B.Y.); liupeipei@4io.org.cn (P.-P.L.); liuxin@4io.org.cn (X.L.); yinqunjian@4io.org.cn (Q.-J.Y.); chenxuyang@4io.org.cn (X.-Y.C.); 2 Guangxi Laboratory of Oceanography, Beihai 536015, China; 3 Guangxi Key Laboratory of Beibu Gulf Marine Resources, Environment and Sustainable Development, Fourth Institute of Oceanography, Ministry of Natural Resources, Beihai 536015, China; 4 Observation and Research Station of Coastal Wetland Ecosystem in Beibu Gulf, Ministry of Natural Resources, Beihai 536015, China; 5 School of Resources, Environment and Materials, Guangxi University, Nanning 530004, China

**Keywords:** gut symbiont, *Candidatus* Hepatincolaceae, snapping shrimp, metagenome-assembled genome, metabolic reconstruction, marine invertebrate, seagrass bed ecosystem

## Abstract

*Candidatus* Hepatincolaceae is a poorly characterized family of obligate Alphaproteobacterial symbionts that are widely detected in ecdysozoans. They were previously assumed to play a nutrient-scavenging role in the gut lumen. In this study, two high-quality metagenome-assembled genomes (MAGs, 1.39 Mb and 1.48 Mb in size) were recovered from the gut of the snapping shrimp *Alpheus brevicristatus* via metagenomic sequencing. Phylogenetic and whole-genome similarity analyses confirm that these two MAGs represent two novel, undescribed genera within the family *Ca*. Hepatincolaceae. Metabolic reconstruction reveals that they not only retain the canonical nutrient-scavenging pathways conserved across all Hepatincolaceae members, but also encode previously undocumented functional modules for antioxidant defense, vitamin B_1_ and B_2_ biosynthesis, and short-chain fatty acid production. They maintain a high oxygen-affinity cytochrome bd terminal oxidase to thrive in the anoxic gut microenvironment. Consistent with their symbiotic lifestyle, their genomes exhibit typical signatures of reductive evolution, such as reduced genome size, low GC content, and gene loss in amino acid and nucleotide de novo biosynthesis pathways. This study presents the first reported high-quality genomes of marine *Ca*. Hepatincolaceae symbionts, which are predicted to possess multiple metabolic functions extending beyond nutritional mutualism.

## 1. Introduction

Host-associated symbiotic bacteria represent critical modulators of organismal fitness across nearly all animal lineages [1]. Rather than acting as passive residents on host tissues, these symbionts substantially expand the metabolic capacity far beyond the functional repertoire encoded solely by the host genome. They mediate a diverse array of host functions, ranging from nutrient acquisition and xenobiotic detoxification to immune regulation and stress resistance [2]. For marine invertebrates that occupy dynamic, fluctuating coastal habitats, symbiotic bacteria are increasingly recognized as a key determinant of host adaptability to variable environmental conditions, such as periodic hypoxia, sulfide exposure, and diet heterogeneity in seagrass and mangrove ecosystems [3,4].

Burrowing shrimps from the families Alpheidae, Thalassinidae and Upogebiidae are common components of invertebrate communities associated with seagrass beds [5]. They can modify sediment geomorphology and accelerate benthic elemental cycling through burrowing activity and foraging behavior [6]. The burrowing shrimps *Alpheus macellarius* and *Neaxius acanthus* actively harvest seagrass litter in their burrows and primarily feed on detrital seagrass leaves [7,8]. Another independent study also verified that benthic microalgae and seagrass detritus serve as the primary dietary sources of snapping shrimps, collectively accounting for 39% of their total food intake [9]. These observations lead to the hypothesis that host-associated symbionts, particularly the gut microbiota of benthic invertebrates in seagrass ecosystems, may enhance host environmental tolerance by selectively enriching functional microbial taxa involved in seagrass detritus degradation. Our earlier 16S rRNA gene amplicon characterization of the gut microbiota of *A. brevicristatus* from Beibu Gulf identified a dominant, unclassified lineage affiliated to Alphaproteobacteria, which accounted for up to 68.7% of the total gut bacterial community [10]. The taxonomic affiliation and functional roles of this highly abundant symbiont, however, remained entirely unresolved prior to the present study.

*Candidatus* Hepatincolaceae (hereafter referred to as Hepatincolaceae) is taxonomically classified as a family within the order Holosporales (Alphaproteobacteria) according to the List of Prokaryotic names with Standing in Nomenclature (LPSN) [11]. The type species *Ca*. Hepatincola porcellionum was first identified in the midgut glands (hepatopancreas) of the terrestrial isopod *Porcellio scaber* [12]. Subsequently, members of this family were widely detected in diverse ecdysozoan animals, including crustaceans (*Armadillidium vulgare*, *Oniscus asellus*, *Trachelipus rathkii*, *Porcellio* spp.), insects (*Labiotermes labralis*), myriapods (*Strigamia maritima*), and tardigrades (*Richtersius* cf. *coronifer*) [13,14,15]. Hepatincolaceae symbionts reside in the host gut lumen, where they establish extracellular associations with host microvilli [12,14]. To date, only six published genomes are currently publicly available, and no member of Hepatincolaceae has been successfully cultivated in vitro. So, current biological understanding of this family remains highly insufficient. Their functional characterization relies predominantly on genomic and metagenomic reconstructions. Hepatincolaceae symbionts are characterized by highly streamlined genomes (~1.2–1.5 Mb), low GC contents (~30%), and limited metabolic capacities, which is consistent with long-term evolutionary adaptation to host-dependent lifestyle. They retain core metabolic pathways for carbohydrate fermentation and ATP generation via substrate-level phosphorylation. Genomic analyses reveal that Hepatincolaceae symbionts may have greater nutritional flexibility than their intracellular counterparts, enabling utilization of various host-derived molecules under anaerobic conditions. So, they are proposed to be nutrient scavengers in the host gut lumen [14,15].

In the present study, we performed shotgun metagenomic sequencing on the gut tissue dissected from an *A. brevicristatus* individual collected from a subtropical seagrass bed in the Beibu Gulf, South China Sea, and successfully recovered two high-quality draft genomes affiliated to the family Hepatincolaceae. Using phylogenetic and comparative metabolic analyses, we resolved their phylogenetic placement within Alphaproteobacteria, and characterized their metabolic potential relative to previously reported terrestrial Hepatincolaceae reference genomes. These results represent the first documented genome of marine Hepatincolaceae symbionts, and genomic analysis suggests that this family may contribute to multiple aspects of host-microbe interactions beyond nutrient scavenging.

## 2. Materials and Methods

### 2.1. Sample Collection and DNA Extraction

A total of nine snapping shrimps *A. brevicristatus* were captured on 8 April 2022 from a shallow seagrass bed in Beihai, Guangxi province, China (21.4322° N, 109.2835° E). The seagrass bed in the intertidal zone is patchily distributed and dominated by the seagrass species *Halophila ovalis*. Collected specimens were transported to laboratory in a tank at 4 °C containing in situ seawater and immediately dissected to collect their gut tissues. All samples were frozen at −80 °C prior to DNA isolation.

The guts of *A. brevicristatus* are dominantly occupied by unclassified Alphaproteobacteria, as reported in our previous study [10]. In order to obtain their draft genomes, the specimen with the highest percentage of targeted bacteria (based on 16S rRNA amplicon analysis) was selected for metagenomic sequencing. The gut sample was cut into small pieces with sterile scissors in advance. Total DNA was extracted using a DNeasy PowerSoil Pro Kit (QIAGEN, Hilden, Germany) following the manufacturer’s protocol. The integrity and purity of the extracted DNA were assessed by 1.5% agarose gel electrophoresis and a Nano-500 spectrophotometer (Allsheng, Hangzhou, China), respectively. DNA concentration was quantified using a Qubit 4.0 Fluorometer (Thermo Scientific, Waltham, MA, USA).

### 2.2. Metagenomic Sequencing, Assembly and Binning

Metagenomic library was constructed from 200 ng of genomic DNA using the TruSeq Nano DNA LT Library Prep Kit (Illumina, San Diego, CA, USA). Paired-end sequencing (2 × 150 bp) was performed on the NovaSeq 6000 platform (Illumina, San Diego, CA, USA) of Shanghai Personalbio Technology Co., Ltd. (Shanghai, China). A total of 32,386,777 paired-end raw reads were generated. The quality of raw reads was initially checked using FastQC v0.12.0 (https://www.bioinformatics.babraham.ac.uk/projects/fastqc/ (accessed on 25 July 2026)). Raw reads were subsequently trimmed using Trimmomatic v0.39 with the following parameters: LEADING: 3, TRAILING: 3, SLIDINGWINDOW: 4:15, and MINLEN: 50 [16]. The filtered reads were then de novo assembled into contigs using MEGAHIT v1.2.9 with the meta-large preset parameter [17]. Metagenome-assembled genomes (MAGs) were recovered using MetaWRAP v1.3.2, which integrated three independent algorithms (MaxBin2, metaBAT2, and CONCOCT) for initial bin extraction [18]. The completeness and contamination level of the retrieved MAGs were estimated using CheckM2 v1.1.0 [19]. High-quality MAGs with completeness ≥ 90% and contamination ≤ 10% were retained for downstream analysis, and taxonomic classification of the MAGs was performed using GTDB-Tk v2.4.0 [20]. Read counts mapped to each MAG and the average sequencing depth of each MAG were calculated using CoverM v0.7.0 [21].

### 2.3. Phylogenetic Analysis Based on 16S rRNA Genes

To clarify the taxonomic positions of the unclassified Alphaproteobacteria, 103 16S rRNA gene sequences from 27 orders within the class Alphaproteobacteria were retrieved from the SILVA SSU 138.2 database. At least three representative sequences were selected from each order to reconstruct the reference phylogenetic tree. Four 16S rRNA gene sequences belonging to the phylum Spirochaetota were used as outgroups. All sequences were aligned using MAFFT v7.505 with the “auto” strategy and normal alignment mode [22]. Gap sites in the aligned matrix were removed using trimAl v1.2rev57 with the “-automated1” command [23]. The maximum likelihood (ML) phylogenetic tree was inferred using IQ-TREE v2.2.0 under the best-fit nucleotide substitution model TPM3 + I + G4, with 1000 ultrafast bootstrap replicates to assess node support [24]. Bootstrap support values > 70% were considered to represent robust phylogenetic placement. Graphical visualization of the phylogenetic tree was performed using the iTOL v7.5 online tool [25]. A second ML tree based on 16S rRNA genes was constructed following the same pipeline, incorporating 41 Rhodospirillales and 39 Holosporales reference taxa to further resolve the phylogenetic placement of our MAGs at lower taxonomic ranks.

### 2.4. Orthology Inference and Phylogenomic Analysis

A total of 29 Rhodospirillales and 19 Holosporales publicly available genomes were downloaded from the NCBI database. Three Gammaproteobacteria genomes were also retrieved and used as outgroups for phylogenomic analysis. Single-copy orthologous genes shared between binned MAGs and all reference genomes were identified using OrthoFinder v2.5.5 [26]. Amino acid sequences of each conserved single-copy gene were aligned using Muscle v3.8.1551 [27]. After ambiguous sites trimming, the curated alignments were concatenated into a partitioned supermatrix using PhyloSuite v2 [28]. The phylogenomic tree was constructed using IQ-TREE v2.2.0 in partition mode, with 1000 ultrafast bootstrap replicates, and the best-fit substitution model for each partition was automatically determined using ModelFinder v2.2.0 [29].

### 2.5. Genome Annotation and Metabolic Prediction

The rRNA and tRNA genes in the assembled MAGs were identified using Barrnap v 0.9 (https://github.com/tseemann/barrnap (accessed on 25 July 2026)) and ARAGORN v1.2.41 [30], respectively. Protein-coding genes (PCGs) were predicted using Prodigal v2.6.3 [31]. For functional annotation, all predicted protein sequences were searched against the NCBI non-redundant protein (NR, 7 February 2024 release ), Swiss-Prot, and Clusters of Orthologous Groups (COGs), using DIAMOND BLASTP v2.1.24 with an e-value cutoff of 1 × 10^−5^ [32]. Conserved protein family domains were identified using the PfamScan tool [17]. KEGG Orthology (KO) assignment and metabolic pathway mapping were performed via the KEGG Automatic Annotation Server (KAAS), employing the bidirectional best-hit method and the default prokaryotic gene reference dataset [33]. Five previously reported Hepatincolaceae genomes were used as references for comparative analysis, and all reference genomes were re-annotated following the exact same pipeline to eliminate analytical bias. The whole-genome average nucleotide identity (ANI) between pairwise genomes was calculated using OrthoANI v0.93.1 [34]. Average amino acid identity (AAI) was also computed using the FastAAI tool [35].

## 3. Results

### 3.1. Draft Genome Features

In total, 9.78 Gb of raw reads were generated from metagenomic sequencing, and 9.07 Gb of clean reads were retained after quality filtering. Metagenomic assembly and binning recovered two draft genomes, designated MS_bin1 and MS_bin2. Since the majority of sequencing reads derived from the host shrimp gut tissue, only 5.90% and 0.60% of the clean reads could be mapped to MS_bin1 and MS_bin2, respectively, corresponding to an average sequencing depth of 197× and 18×. This distribution indicated that MS_bin1 represented a high-abundance dominant symbiont, while MS_bin2 occurred at considerably lower relative abundance in the gut microbial community. The estimated genome sizes of MS_bin1 and MS_bin2 were approximately 1.39 Mb and 1.48 Mb, with low GC contents of 29.2% and 26.2% (Table 1). Although the MAGs were not completely closed, they still exhibited extremely high assembly quality, characterized by near-full completeness (97.21% and 95.20%) and very low contamination levels (0.02% and 0.09%). Additionally, three rRNA genes (5S, 16S, and 23S) were predicted in MS_bin1, while only the 16S rRNA gene was identified in MS_bin2. MS_bin1 and MS_bin2 encoded 1303 and 1419 PCGs, with coding densities estimated at 86.1% and 88.7%. Through database alignment, 1039, 723, 915, 1005, and 740 PCGs from MS_bin1 were successfully annotated against the NCBI NR, Swiss-Prot, COG, Pfam, and KEGG databases, respectively. For MS_bin2, 1170, 822, 1000, 1079, and 804 PCGs were annotated in the corresponding databases.

### 3.2. Taxonomic Classification

**Figure 1:**
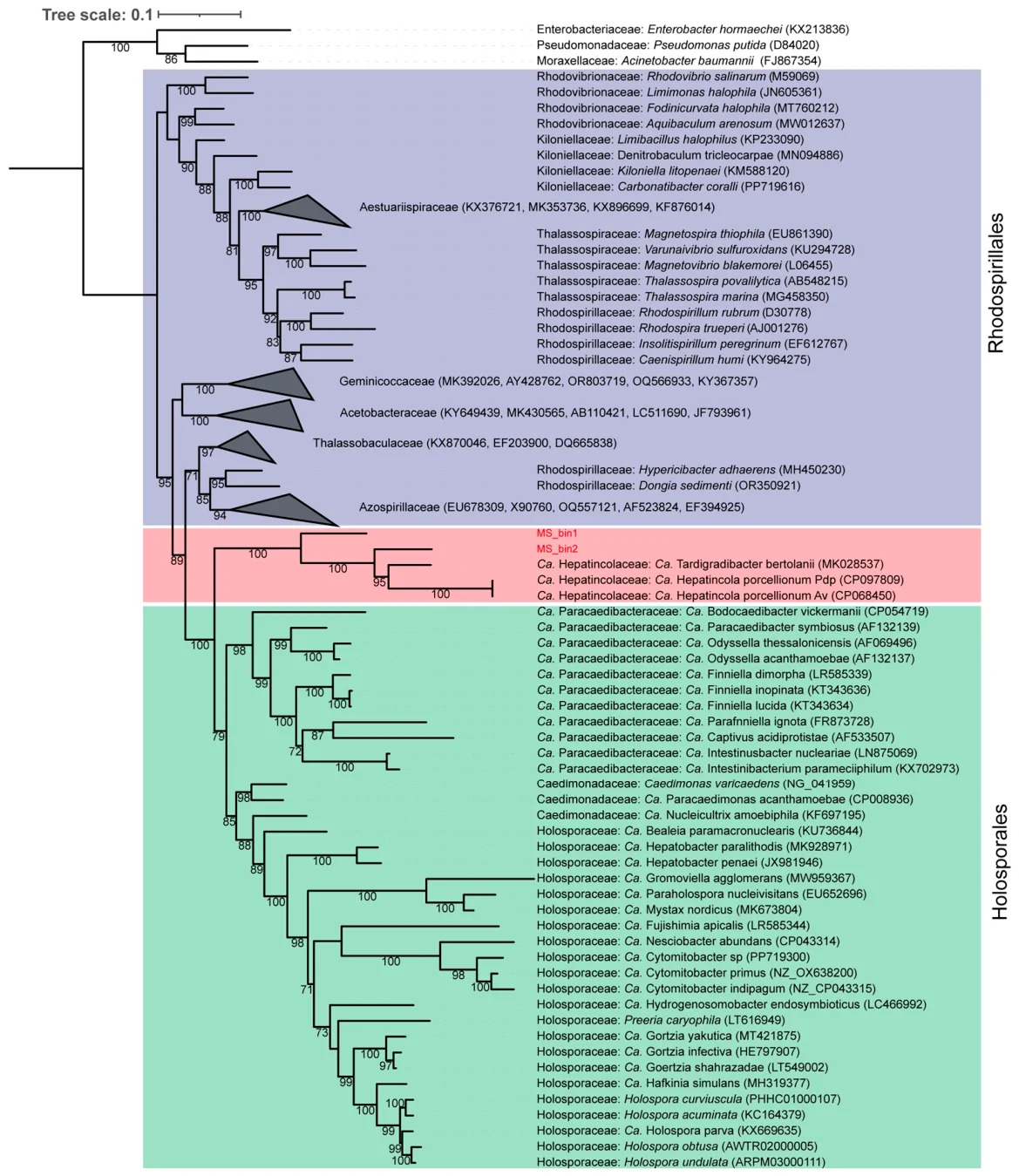
Phylogenetic trees inferred from 16S rRNA genes. Three Gammaproteobacteria species are used as outgroups. The species names in red come from this study. The bacteria of *Ca*. Hepatincolaceae, Holosporales and Rhodospirullales are labeled with red, green and blue backgrounds. All sequences are labeled with their NCBI accession numbers. Nodes with greater than 70% bootstrap support are labeled. Scale bar represents substitutions per site. The black triangle represents a collapsed monophyletic node containing multiple closely related sequences from the same taxon group.

**Figure 2:**
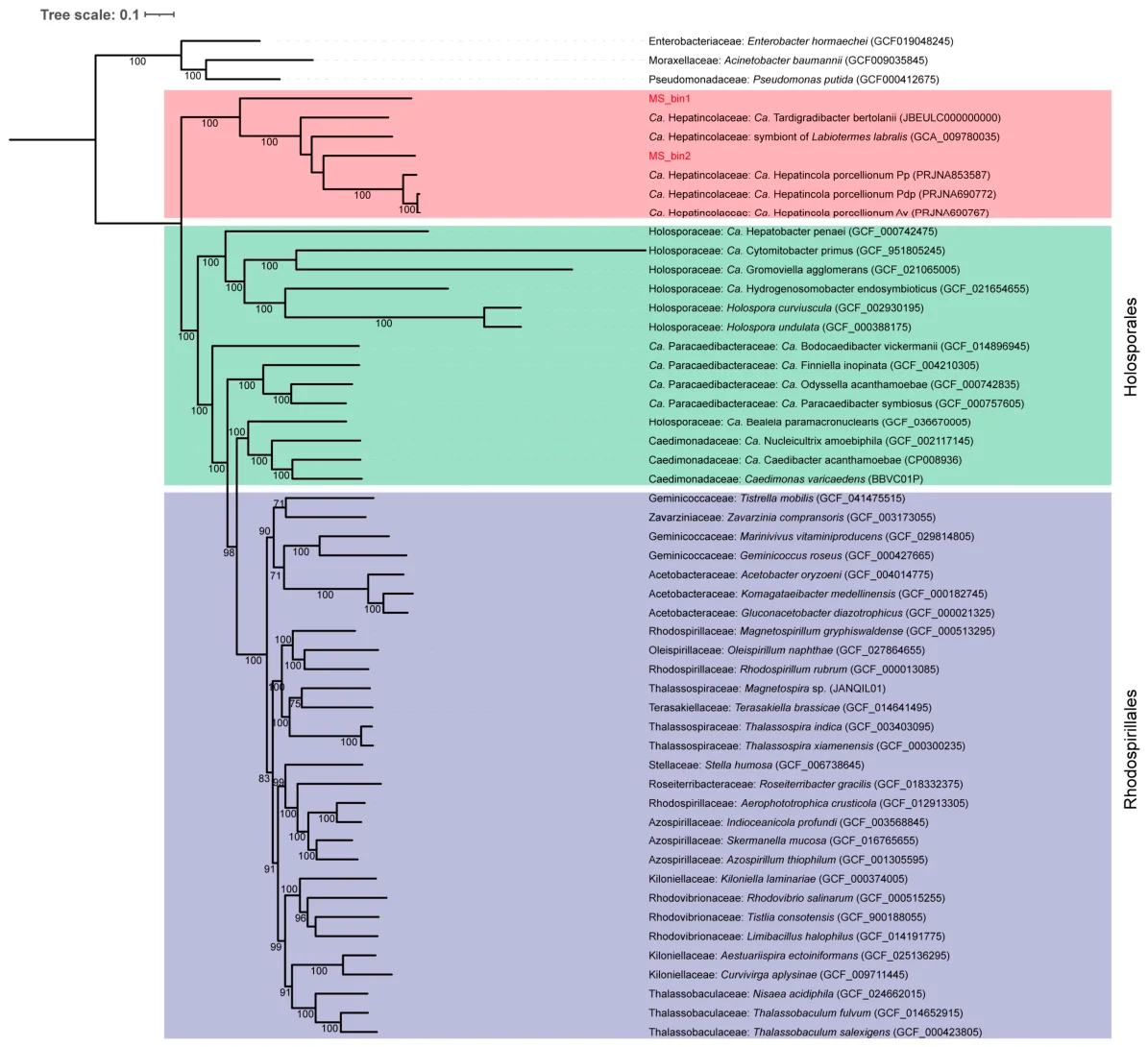
Phylogenetic trees inferred from single-copy orthologous genes. Three Gammaproteobacteria species are used as outgroups. The genome sequences in red come from this study. The bacteria of *Ca*. Hepatincolaceae, Holosporales and Rhodospirullales are labeled with red, green and blue backgrounds. All sequences are labeled with their NCBI accession numbers. Nodes with greater than 70% bootstrap support are labeled. Scale bar represents substitutions per site.

The 16S rRNA gene sequences of MS_bin1 and MS_bin2 were extracted for taxonomic assignment and phylogenetic inference. Their top hits in the NCBI 16S rRNA database were both *Caedimonas varicaedens* strain 221, with sequence identities of 85.01% and 83.81%, respectively. When employing the Genome Taxonomy Database (GTDB) for taxonomic identification, both MS_bin1 and MS_bin2 were classified into the order-level undescribed clade WRAU01. Using 16S rRNA gene sequences from 27 orders of the class Alphaproteobacteria, our initial phylogenetic analysis robustly placed the two MAGs within a well-supported monophyletic clade that clustered with *Ca*. Hepatincola porcellionum and the symbiont of *Cherax quadricarinatus* (Figure S1). However, a known taxonomic discrepancy exists in current public databases: *Ca*. *H. porcellionum* is provisionally classified as Rickettsiales incertae sedis in the SILVA v138.2 database, but is formally assigned to the order Holosporales in the LPSN database. To ensure the validity of family-level taxonomic classification of our MAGs, reference 16S rRNA sequences of Holosporales and Rhodospirillales were downloaded from the LPSN database for refined phylogenetic analysis. The resulting phylogenetic tree confirmed that MS_bin1 and MS_bin2 formed a distinct monophyletic clade with all published members of the Hepatincolaceae family, supported by a 100% bootstrap value (Figure 1). Within the order Holosporales, the family *Ca*. Paracaedibacteraceae formed a sister group to the families Caedimonadaceae and Holosporaceae, while Hepatincolaceae occupied an early-branching position relative to these three families. Additionally, the Rhodospirillales lineages diverged from their common ancestor earlier than the Holosporales lineages. A maximum-likelihood phylogenomic analysis, constructed from 71 single-copy orthologous genes shared across 50 genomes, further confirmed the stable placement of our binned MAGs within the Hepatincolaceae family (Figure 2). However, the relative divergence order of Rhodospirillales compared to Holosporales differed between the 16S rRNA gene-based phylogenetic tree and the phylogenomic tree: Rhodospirillales diverged later than Holosporales in the phylogenomic tree, resulting in an opposite branching topology.

### 3.3. General Genome Comparison

**Figure 3:**
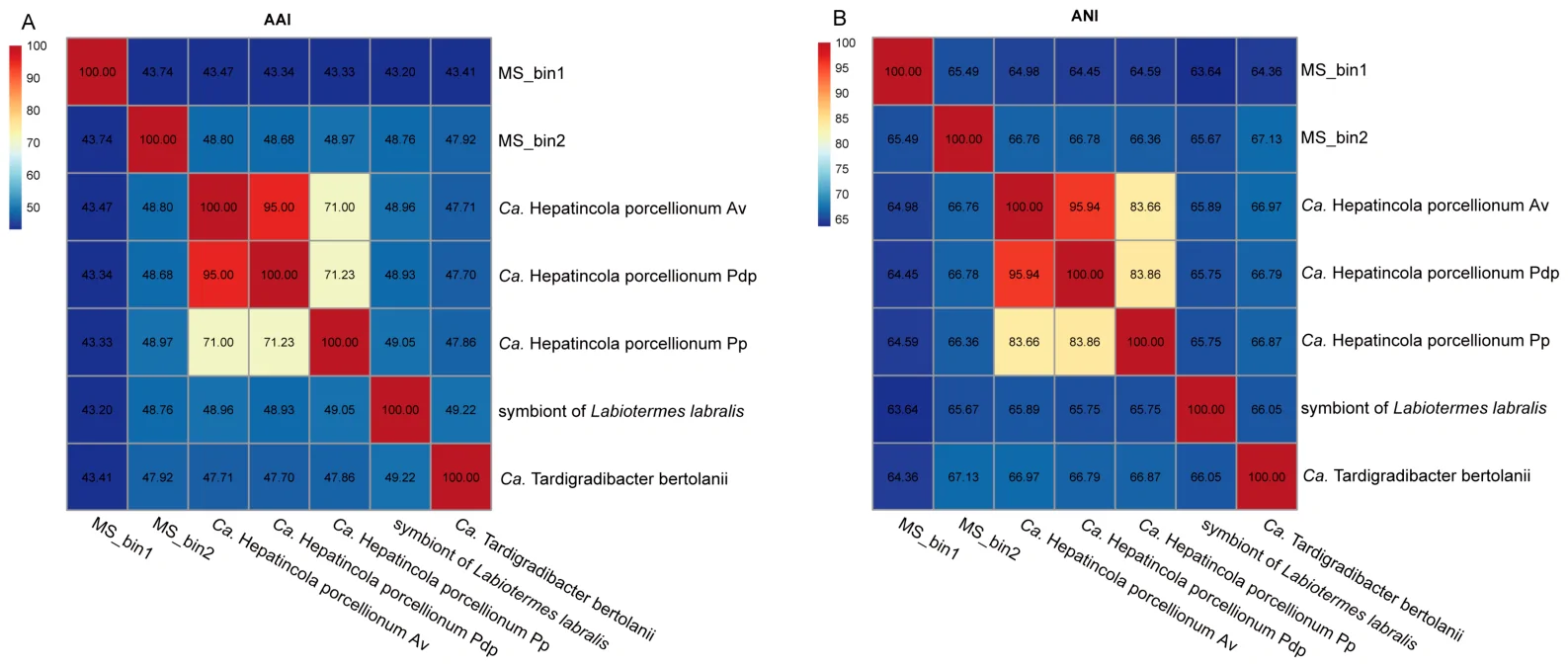
Heatmaps of genomic similarity between metagenome-assembled genomes and related taxa. (**A**) Heatmap of average amino acid identity (AAI); (**B**) heatmap of average nucleotide identity (ANI). Both rows and columns correspond to the tested genomes; the cell color represents the similarity value obtained from pairwise comparison of corresponding genomes. The values on the right of the color bar indicate the range of similarity percentages.

Pairwise AAI between MS_bin1 and MS_bin2 was 43.74%, and the two MAGs showed 43.20–48.97% AAI to all publicly available Hepatincolaceae genomes (Figure 3). Meanwhile, the ANI only ranged from 63.64% to 67.13%. Both metrics were far below the widely accepted thresholds for prokaryotic genus-level (60–65% AAI) and species-level (95% ANI) demarcation, respectively [36,37]. So, our retrieved MAGs represent two previously undescribed species belonging to two novel genera within the Hepatincolaceae family.

**Figure 4:**
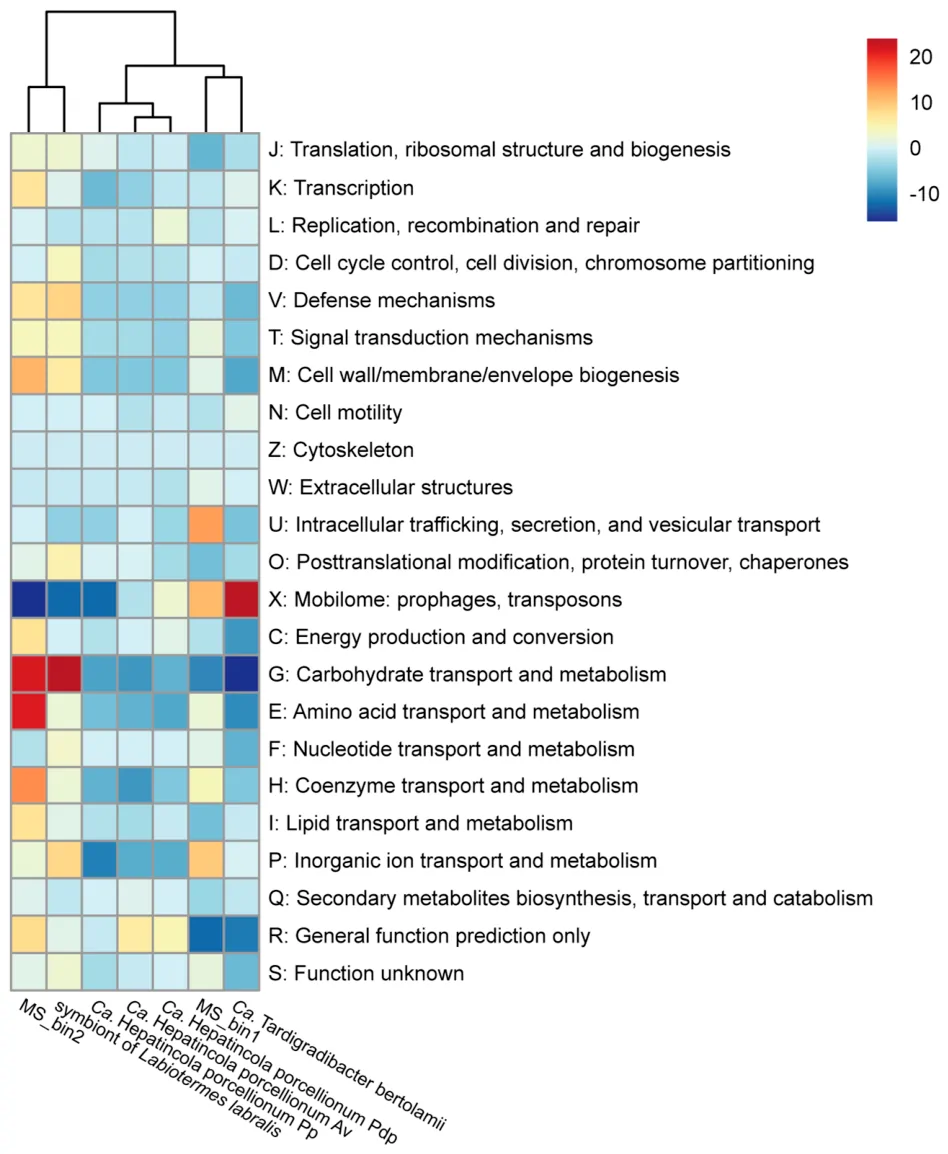
Heatmap showing the COG profiles of *Ca*. Hepatincolaceae genomes. Hierarchical clustering of COG categories (columns) was performed using Euclidean distance with complete linkage, and color intensity indicates mean subtraction normalized relative abundance. Heatmap is generated using R version 3.6.3.

COG functional annotation revealed that core essential pathways including translation, transcription and DNA replication/repair were largely preserved across all seven Hepatincolaceae genomes, which further validated the near-complete assembly quality of our two MAGs (Figure 4). However, distinct functional profiles were observed between the two marine MAGs. MS_bin2 contained significantly expanded gene sets involved in carbohydrate and amino acid transport and metabolism, supporting its putative role as a digestive symbiont that participates in complex polysaccharide degradation and host nutrient supply. MS_bin1 encoded the largest repertoire of intracellular secretion system components, as well as abundant mobile genetic elements (prophages/transposons), indicating high genome plasticity and specialized mechanisms for host–symbiont interaction. Hierarchical clustering based on COG category abundance showed that MS_bin2 clustered adjacent to the gut symbiont of *Labiotermes labralis*, while MS_bin1 grouped closely with *Ca*. Tardigradibacter bertolamii, reflecting their high degree of functional similarity to their corresponding terrestrial symbiont relatives. Finally, pan-genome analysis of all seven Hepatincolaceae genomes identified a core set of 622 shared orthogroups, and 84 orthogroups that were unique to the two marine MAGs and completely absent in reported terrestrial and freshwater representatives (Figure S2).

### 3.4. Carbohydrate and Energy Metabolism

The metabolic networks of MS_bin1 and MS_bin2 were reconstructed based on KEGG annotation results (Figure 4). Both MAGs retain the vast majority of core reactions involved in carbohydrate and energy metabolism, including glycolysis and the non-oxidative phase of the pentose phosphate pathway, but lack a complete tricarboxylic acid (TCA) cycle. Based on genomic annotation, they can convert glucose to pyruvate, and generate a net yield of two ATP and two NADH molecules through substrate-level phosphorylation. Pyruvate is further oxidized to acetyl-CoA, which can be converted to acetate via the phosphotransacetylase-acetate kinase (PAT-ACK) pathway, with acetyl-phosphate acting as the metabolic intermediate. Notably, MS_bin1 carries a classic ethanol fermentation pathway that converts acetyl-CoA into ethanol. In contrast, MS_bin2 can reduce pyruvate to lactate via lactate dehydrogenase. Accordingly, lactate, ethanol, and acetate are predicted to be the major end products of anaerobic fermentation in these two symbiont strains. Both MS_bin1 and MS_bin2 encode complete phosphotransferase systems (PTS) responsible for the transport of glucose and cellobiose; MS_bin2 also possesses an additional dedicated PTS for fructose uptake. In addition, both genomes encode oligo-1,6-glucosidase. This enzyme specifically hydrolyzes (1,6)-α-D-glucosidic linkages in common oligosaccharides (such as isomaltose and α-limit dextrin) produced from starch and glycogen degradation by α-amylase, directly releasing free glucose for downstream metabolic utilization. Furthermore, both MS_bin1 and MS_bin2 are capable of synthesizing propanoate and succinate, with 2-oxoglutarate and oxaloacetate serving as their corresponding precursors, respectively.

Both MS_bin1 and MS_bin2 carry a highly streamlined aerobic respiratory chain. The encoded NADH:quinone oxidoreductase oxidizes the NADH generated from glycolysis back to NAD^+^, while pumping protons across the cell membrane to establish the transmembrane proton motive force required for ATP synthesis. MS_bin1 encodes the flavoprotein subunit (SdhA) and the iron–sulfur subunit (SdhB) of succinate dehydrogenase, but no sequences annotated as the membrane-anchored SdhC and SdhD subunits were retrieved in the homology searches against the NR and KEGG databases. However, the conserved domain scan successfully identified one candidate *sdhC* gene containing a complete Sdh_cyt (succinate dehydrogenase/fumarate reductase transmembrane subunit) domain. The predicted SdhC protein contains 136 amino acids with three transmembrane helices. Finally, the cytochrome bd oxidase (CydAB) represents the sole terminal oxidase in both symbiotic strains.

### 3.5. Nucleotide, Amino Acid and Coenzyme Metabolism

**Figure 5:**
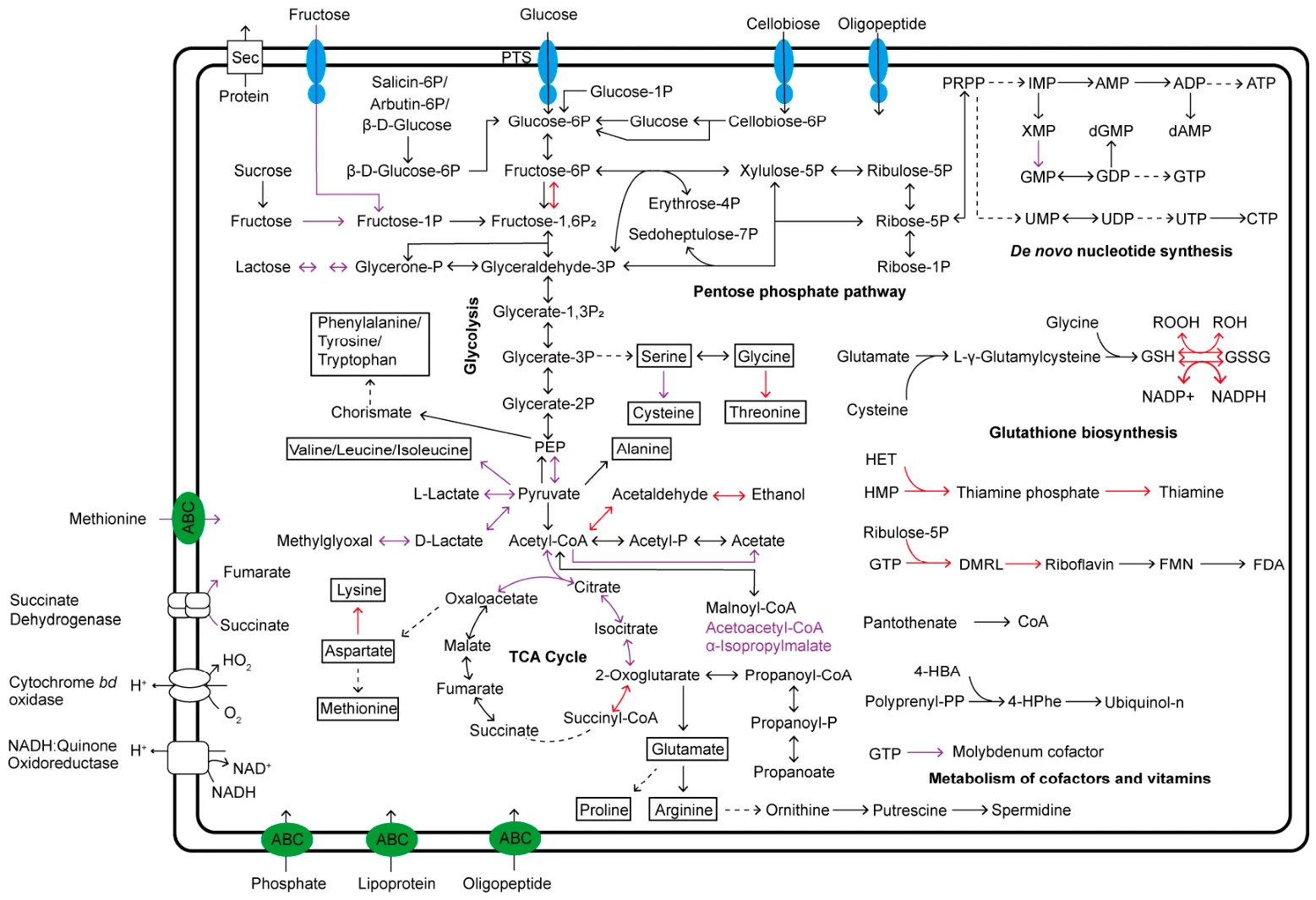
The inferred metabolic network of metagenome-assembled genomes. The unique metabolic pathways of MS_bin1 are marked with red lines, those exclusive to MS_bin2 are indicated with purple lines, and shared metabolic pathways are represented by solid black lines. Absent metabolic pathways are shown in dotted lines.

Neither MS_bin1 nor MS_bin2 can synthesize purines and pyrimidines de novo from phosphoribosyl pyrophosphate (PRPP) (Figure 5). However, they retain the full gene set required for the nucleotide salvage pathway, with genes encoding adenine phosphoribosyltransferase (APRT), xanthine phosphoribosyltransferase (XPRT), and uracil phosphoribosyltransferase (UPRT). Both MAGs can synthesize alanine from pyruvate, and produce glutamate and arginine using 2-oxoglutarate as the precursor. MS_bin2 also retains the biosynthetic pathway required for valine, leucine and isoleucine production from pyruvate. Aside from these conserved routes, both MAGs have lost key functional genes required for the de novo biosynthesis of other essential amino acids. They are predicted to acquire free amino acids by degrading exogenous oligopeptides and lipoproteins, which are first transported into the cell via corresponding ABC transporters. In addition, MS_bin2 encodes a specific methionine ABC transporter, enabling it to directly uptake free methionine distributed in the host gut. Furthermore, both MS_bin1 and MS_bin2 retain the canonical polyamine biosynthesis pathway, which uses ornithine as the precursor to synthesize spermidine. Most notably, MS_bin1 can synthesize the key antioxidant molecule glutathione (GSH) using glutamate, cysteine, and glycine as raw substrates. It also encodes NADPH-dependent glutathione reductase (GR), which reduces oxidized glutathione (GSSG) back to the biologically active reduced form. In addition, MS_bin1 encodes glutathione peroxidase (GPx). Using GSH as the hydrogen donor, GPx can directly catalyze the clearance of various intracellular reactive oxygen species, effectively blocking the propagation chain of oxidative damage. This genomic feature indicates that MS_bin1 possesses a complete antioxidant defense system.

MS_bin1 carries a complete thiamine salvage pathway, which acts as the core metabolic pathway for bacteria to maintain intracellular thiamine (vitamin B_1_) homeostasis. With this pathway, MS_bin1 can efficiently utilize pre-existing environmental thiamine and its degradation intermediates in the gut, rather than relying entirely on energy-costly de novo thiamine synthesis. MS_bin1 also contains nearly all key genes in the riboflavin (vitamin B_2_) biosynthesis pathway, which produces riboflavin from GTP and ribulose-5-phosphate. The only missing gene in the pathway is the one encoding 5-amino-6-(5-phospho-D-ribitylamino) uracil phosphatase. However, MS_bin1 encodes multiple copies of HAD (haloacid dehalogenase) family phosphatase. This enzyme can functionally compensate for this unannotated dephosphorylation step, resulting in a complete thiamine biosynthetic capacity.

## 4. Discussion

In this study, we recovered two high-quality Hepatincolaceae MAGs from the gut of *A. brevicristatus*, which exhibited typical features of symbionts. Their reduced genome sizes (~1.4 Mb) and low GC contents are highly consistent with reported terrestrial Hepatincolaceae relatives [14,15]. The characteristic patterns of reductive genome architecture and biased nucleotide base composition are widely observed in obligate host-associated symbiotic bacteria, as a consequence of long-term reductive evolution [38]. The current evidence demonstrates that Hepatincolaceae maintain an extracellular attachment to the host intestinal microvilli [12,39]. However, the gut-associated MS_bin1 and MS_bin2 are unlikely to possess the capacity for independent free-living survival. Because extensive gene loss has occurred in their central metabolic pathways, including genes participating in de novo nucleotide synthesis, essential amino acid biosynthesis, and the TCA cycle, they presumably represent obligate ectosymbionts with highly specialized adaptations to the gut microenvironment.

Previously characterized terrestrial Hepatincolaceae members are predicted to perform basic nutrient-scavenging functions [14,15]. Similarly, both MS_bin1 and MS_bin2 encode transporters and processing enzymes for multiple sugars and other carbon sources, enabling them to utilize the digestive products of microalgae and seagrass detritus. To thrive in the oxygen-deficient gut environment, the marine Hepatincolaceae symbionts employ high oxygen-affinity cytochrome bd oxidase as their terminal respiratory enzyme, and appear to have lost the oxygen-sensitive, low-affinity cytochrome *c* oxidase [40]. This allows them to stay metabolically active under low-oxygen gut conditions, and to readily outcompete other resident gut bacteria.

Metabolic reconstruction suggests that our two marine symbionts may have evolved novel functional capabilities, which are absent or incomplete in their terrestrial relatives. MS_bin1 harbors a complete GSH biosynthesis pathway, enabling it to perform the full GSH redox cycle. GSH is a pleiotropic metabolite that directly scavenges reactive oxygen species (ROS), nitric oxides and carbon radicals [41]. Animals in the intertidal zone are frequently exposed to tide-driven and emersion-associated environmental stressors, such as desiccation, acute temperature oscillation, hypoxia, salinity fluctuations, ultraviolet (UV) radiation, and hydrogen sulfide toxicity [42]. These environmental stressors can trigger intestinal oxidative stress and subsequent tissue damage in shrimp [43,44,45]. The symbiont MS_bin1 is genotypically equipped to alleviate host oxidative stress by scavenging excess ROS in the shrimp gut. The antioxidant glutathione also enables symbionts to successfully colonize and reproduce in the host gut [46]. By mixed acid fermentation, both marine MAGs can produce short-chain fatty acids (SCFAs), including acetate, propanoate and succinate. Usually, marine crustaceans cannot synthesize these SCFAs themselves. The gut microbiome-derived SCFAs not only act as direct energy sources for gut epithelial cells, but also strengthen intestinal barrier integrity, inhibit the colonization of opportunistic pathogens, and help maintain gut immune homeostasis [47,48,49].

We also identified complete thiamine (vitamin B_1_) and riboflavin (vitamin B_2_) biosynthetic pathways in the MS_bin1 genome. Thiamine commonly serves as an indispensable cofactor for key enzymes in glucose and energy metabolism [50], while riboflavin acts as the obligate precursor of flavin adenine dinucleotide (FAD) and flavin mononucleotide (FMN), which are universally required for redox reactions and energy production in animals [51]. As essential metabolites, vitamins cannot be synthesized by the host, and must be obtained exogenously through dietary intake or gut symbiotic microbiome biosynthesis [47]. The results suggest that our new Hepatincolaceae symbiont may act as an important vitamin provider for its shrimp host, contributing to host nutritional supplementation.

Based on genomic analysis, our results reveal that the marine Hepatincolaceae–shrimp symbiosis described here may represent a far more integrated, multi-functional partnership than previously assumed. These symbionts are more than commensal associates predicted to assist the host in nutrient scavenging. They are genomically annotated as versatile partners that putatively confer three benefits on the host: oxidative stress resistance, immune homeostasis regulation, and essential nutrient supplementation. Significantly, all functional inferences are based on genomic predictions and need experimental validation in the future. Then, the sample size for dominant bacterium screening is small, which may limit the generalizability of our findings. Despite these limitations, the genomic resources and metabolic networks generated in this study establish a foundational framework for future experimental validation of Hepatincolaceae–host interactions.

## 5. Conclusions

Two high-quality draft genomes were recovered from the gut of the snapping shrimp *A. brevicristatus* collected from a subtropical seagrass bed in the Beibu Gulf. Phylogenetic analysis confirms that these two shrimp intestinal bacteria represent previously undocumented deep-branching lineages within the family *Ca*. Hepatincolaceae. They are supposed to be obligate symbionts with reduced genome and extensive gene loss in the de novo nucleotide synthesis, essential amino acid biosynthesis, and the TCA cycle. To adapt to the gut environment, they retain the capacity to ferment various sugars and the electron transport chain adapted to low-oxygen condition. Genomic analysis suggests that one of the gut symbionts may possess functional capability far beyond the narrow nutrient-scavenging role previously attributed to the family Hepatincolaceae. This functionally versatile symbiont encodes complete antioxidant defense systems, thiamine and riboflavin provisioning pathways, and short-chain fatty acid biosynthesis modules that likely contribute to host environmental stress tolerance and nutritional supplementation. Our findings substantially expand the known functional diversity of the family *Ca*. Hepatincolaceae.

## Figures and Tables

**Table 1:** General features of metagenome-assembled genomes and reference genomes.

	MS_bin1	MS_bin2	*Ca*. Hepatincola Porcellionum Av [14]	*Ca*. Hepatincola Porcellionum Pdp [14]	*Ca*. Hepatincola Porcellionum Pp [14]	*Ca*. Tardigradibacter Bertolanii [15]	Symbiont of *Labiotermes labralis* [15]
Host	*Alpheus brevicristatus*	*Alpheus brevicristatus*	*Armadillidium vulgare*	*Porcellio dilatatus petiti*	*Porcellionides pruinosus*	*Richtersius* cf. *coronifer*	*Labiotermes labralis*
Genome size (bp)	1,392,888	1,481,805	1,340,542	1,328,361	1,284,105	1,332,542	1,422,127
GC content (%)	29.2	26.2	29.5	29.4	31.6	30.3	33.6
No. of scaffolds	37	20	1	17	1	15	17
N50 (scaffolds) (bp)	113,108	162,790	1,340,542	200,054	1,284,105	996,816	130,920
Protein-coding genes	1303	1419	1187	1104	1139	1180	1378
Coding density (%)	86.1	88.7	86.1	85.9	86.8	81.9	91.0
CheckM2 completeness (%)	97.21	95.20	96.98	98.63	96.01	97.52	99.02
CheckM2 contamination (%)	0.02	0.09	0.01	0.01	0.01	0.02	0.09
rRNA	3	1	6	2	6	6	0
tRNA	33	31	32	30	32	36	34

## Data Availability

The raw data and genome sequences from this study have been deposited at NCBI GenBank with the project accession number PRJNA1210165.

## Supplementary Materials

The following supporting information can be downloaded at: https://www.mdpi.com/article/10.3390/microorganisms14081864/s1, Figure S1: Phylogenetic placement of metagenome-assembled genomes within the Alphaproteobacteria class. Gray wedges represent monophyletic groups of a particular taxonomy. All sequences are labeled with their NCBI accession numbers. Nodes with greater than 70% bootstrap support are labeled. Scale bar represents substitutions per site. Figure S2: Upset plot showing the number of shared orthogroups among Hepatincolaceae genomes. The numbers above each bar indicate the number of orthogroups shared by a specific intersection of genomes. The set sizes indicate the number of orthogroups in each genome.
